# Book Notices

**Published:** 1878-03-20

**Authors:** 


BOOK NOTICES.

---

THE PRACTITIONER'S HAND BOOK OF TREATMENT; or, the Principles of Therapeutics. By J Milner Fothergill, M.D., member of the Royal College of Physicians of London, etc. Philadelphia: Henry C. Lea, 1877.

Dr. Fothergill attempts, in this work, to explain the *rationale* of therapeutical measures generally employed by the enlightened English practitioner. In this he succeeds. He gives an excellent picture of the more advanced methods of treatment as taught and enforced by our English brethren. Dr. F., in detailing his plan of the work, remarks: "First, the physiology of each subject is given, then the pathology is reviewed, so far as they bear upon the treatment; next, the action of remedies is examined, after which their practical application in concrete prescriptions is furnished." The author does not give undue attention to new remedies, but seeks to "analyze and elucidate the *modus operandi* of the measures in common use." His design "is to furnish to the practitioner reasons"—which the practitioner must exercise if he conscientiously strives to be a competent and useful physician—"for the faith that is in him, and is"—which all, both old and young, need—"a work on medical tactics for the bed-side." The author has spent nine years in gathering materials for his work, and the time he has devoted to this purpose bears most excellent fruitage. It is a work we have perused with more than ordinary interest—one that has afforded us an ample amount of information of practical importance. We hesitate not to recommend it to the active practitioner as a work capitally suited to his wants, and one he will read with pleasure, no less than practical advantage.

THE EAR: Its Anatomy, Physiology, and Diseases. A Practical Treatise for the use of Medical Students and Practitioners. By Charles H. Burnett, A.M., M.D., Aural Surgeon to the Presbyterian Hospital, etc. With Eighty-seven Illustrations. Philadelphia: Henry C. Lea, 1877.

Otology has made great advances of late years; hence, the appearance of this work is not only timely, but necessary. In view of the frequency of calls made upon the practitioner, by patients, with diseases of the ear, the study of these becomes a matter of the greatest importance. He can no longer escape the odium of ignorance of these diseases, when a work so satisfactory as the one before us is within his reach. Knowledge is power no less in practice than in the forum or on the battle-field. Knowledge will tell; and the practitioner who will devote his time in attaining medical knowledge, to be found in works like the one above, will excel in his vocation. Dr. Burnett has accomplished his undertaking in a most admirable manner. He is abreast of the times, and gives the profession a most useful and delightful work—one, we trust, no reader of the Record will fail to purchase and study.

Recognition and Management of the Gouty State in Diseases of the Skin. By L. Duncan Bulkley, A.M., M.D., Physician to the Skin Department of Demilt Dispensary, N. Y., etc.

A paper of much interesting matter, presenting new and practical points connected with the relation existing between the gouty state and skin diseases.

On the so-called Eczema Marginatum of Hebra-Tinia Cercenata as observed in America. A Clinical Study. By L. Duncan Bulkley, A.M., M.D., Physician to Skin Diseases, etc. Also, Eczema and Psoriaosis. By the same author.

Heart clots: A Report of Three Cases, and the Etiology, Diagnosis, Prognosis, and Treatment of Cardiac Thrombosis, based on an Analysis of Sixty-eight Cases, and Physiological Experiments. By Martin L. James, M.D., Lecturer on Practice of Medicine, Medical College of Virginia.

This is a paper of interest upon a subject little understood.

Transactions of the Eighth Annual Session of the State Medical Society of Virginia, held in Petersburg, October, 1877.
1877.

This is a very able and highly creditable document. As a native Virginian, we confess to a feeling of pride when we see these transactions replete with instructive and valuable papers from medical gentlemen, many of whom we know personally, as men at the very top of the profession in the Union, a fact well exhibited in the learned and instructive articles contained in this volume. We regret that we have not space to review their papers in detail. The able address of President James L. Cabell opens the volume; then follow the instructive and interesting papers of Dr. W. C. Randolph, on "The Study of Medicine;" Dr. Ellzey, on "The Application of Chemistry in Medico-Legal Science;" Dr. Preston, on "Advances in Obstetrics;" Dr. Apperson, on "Advances in the Practice of Medicine;" Dr. Joynes, on "Advances in Hygiene and Public Health;" Dr. Wellford, on "Poisoning by Custards and Ice Creams;" Dr. Page, on "Epidemic Zymotic Diseases of Animals;" Dr. James, on "Heart-clots;" Dr. Selden, on "Fractures of the Neck of the Femur Within the Capsule;" Dr. Chancellor, on "Iodoform as a Local Remedy;" closing with an interesting discussion on "Instrumental Labor," and "Report of the Necrological Committee."

The following are the officers elect for the present year:

J. H. Claiburn, M.D., President.
Oscar Wiley, M.D., First Vice-President.
J. R. Goodwin, M.D., Second Vice-President.
J. E. Parrish, M.D., Third Vice-President.
W. C. Randolph, M.D., Fourth Vice-President.
W. S. Love, M.D., Fifth Vice-President.
J. S. Wellford, M.D., Sixth Vice-President.
L. B. Edwards, M.D., Secretary and Treasurer.
C. Tompkins, M.D., Corresponding Secretary.

P.

Transactions of the Medical Society of the State of North Carolina, at the May meeting in Salem, 1877.

We have just discovered that a copy of the above, kindly sent us by a medical friend sometime ago, though reviewed and sent to the printer, was unintentionally overlooked.

There was a good attendance at the last meeting. An address of welcome, by Colonel Pattison, was handsomely responded to by the President, Dr. Foote.

A number of interesting cases were reported and discussed.

Dr. Holmes, chairman of the Committee on Schools, submitted a scathing criticism on the "So called Medical College" in the county of Robeson. The principal, Hector McLean, and a young, incompetent man, his son, constitute the entire faculty, and have been issuing diplomas to medical students.

An interesting and somewhat elaborate paper on Epilepsy was presented by Eugene Grissom, M.D., Superintendent of the Insane Asylum of North Carolina. Also, valuable reports by W. W. Lane, M.D., A. G. Carr, M.D.

The valedictory address was delivered by Dr. George A. Foote. It was able and practical.

The present officers are as follows:

Dr. R. L. Payne, President.
Dr. F. M. Rountree, First V. P.
Dr. Richard Anderson, Second V. P.
Dr. S. B. Flowers, Third V. P.
Dr. L. A. Smith, Fourth V. P.
Dr. Julian Picot, Secretary.
Dr. A. G. Carr, Treasurer.
Dr. W. T. Ennett, Orator.

The next meeting of the society will be held at Goldsboro, on the 14th of May next.

---

Several interesting pamphlets cannot be noticed in present issue for want of space.

